# Supplementary figures and images for: Benchmarking Bacterial Promoter Prediction Tools: Potentialities and Limitations
Source: mSystems. 2020 Aug 25;5(4):e00439-20. doi: 10.1128/mSystems.00439-20 (PMC7449607; doi:10.1128/mSystems.00439-20)

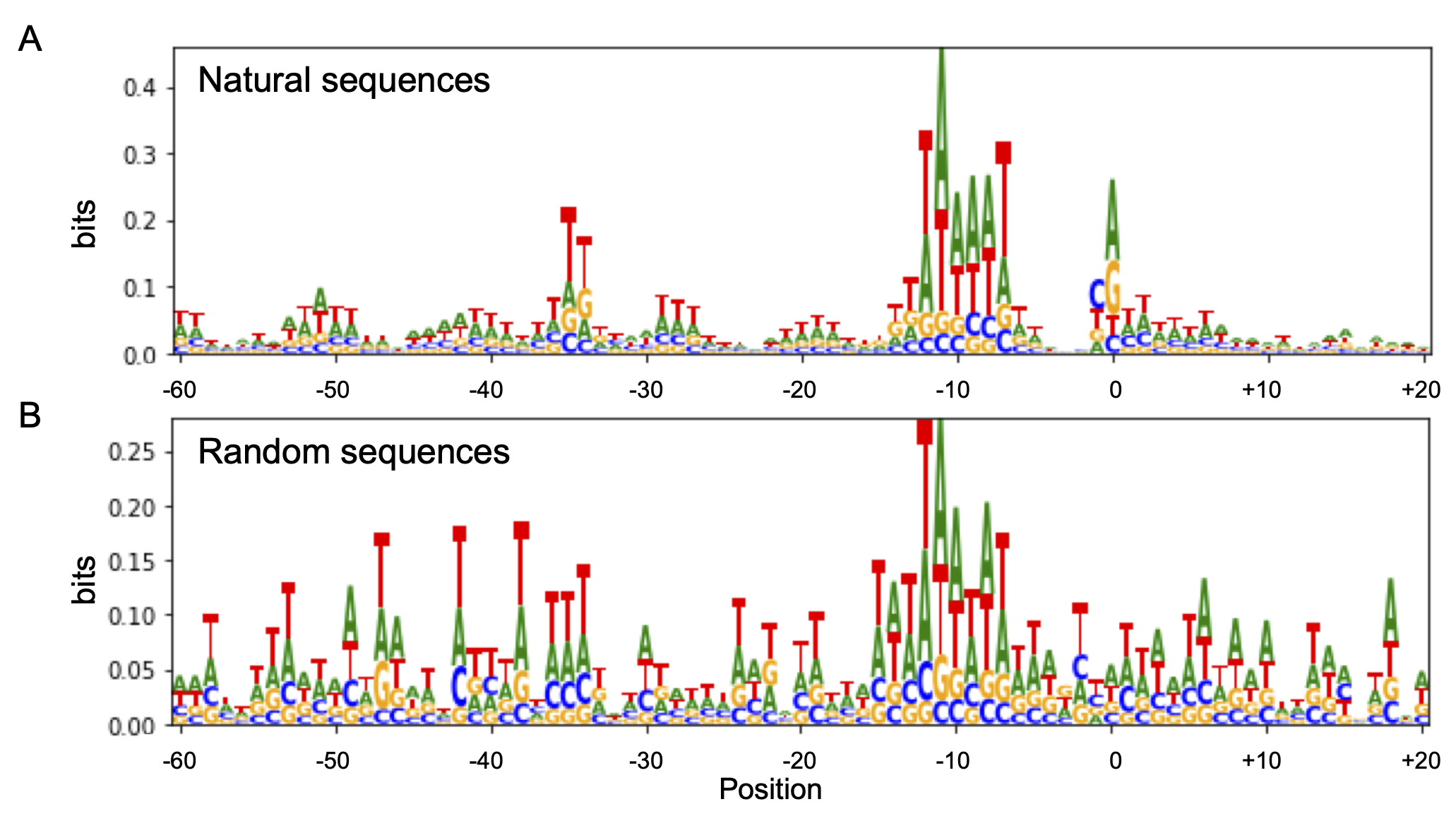

Supplement: FIG S1 [file mSystems.00439-20-sf001.tif]
